# A shared reading intervention: Changing perceptions of caregivers in a semi-rural township

**DOI:** 10.4102/sajcd.v70i1.948

**Published:** 2023-01-26

**Authors:** Tarryn Coetzee, Sharon Moonsamy, Joanne Neille

**Affiliations:** 1 Department of Speech-Language Pathology and Audiology, School of Human and Community Development, University of the Witwatersrand, Johannesburg, South Africa

**Keywords:** literacy, shared-reading, early intervention, caregiver training, caregiver perceptions, South Africa, emergent literacy development

## Abstract

**Background:**

Many caregivers from low-middle income (LMI) households consider that preschool children are too young for shared book reading. Thus, many caregivers are unaware of their potentially powerful role in their children’s emergent literacy and communication.

**Objectives:**

To describe (1) caregivers’ perceptions of shared reading, (2) caregivers’ perceptions of barriers to shared reading and (3) changes in these perceptions following a short intervention.

**Method:**

A qualitative methodology was used to understand the perceptions of 40 caregivers from a semi-rural South African township. Two semi-structured interviews were conducted before and after intervention. The intervention was a short training video about shared reading.

**Results:**

Caregivers described the unfamiliar reading culture and viewed reading as an educational activity that they knew little about. Barriers to shared reading included lack of time, few reading materials and low levels of literacy or lack of exposure to this type of activity. Following the intervention, they acknowledged the importance of shared reading, described growing confidence in their shared reading abilities and closer relationships with their children.

**Conclusion:**

Speech-language therapists (SLTs) have a pivotal role to play in caregiver training of emergent literacy skills and can make a marked impact in guiding caregivers’ shared reading. A short video-based intervention can alter caregiver perceptions and practices, which may be the first step in changing behaviours.

**Contribution:**

The study provides an example of a simple and cost-effective intervention that changed caregiver perception and caregivers’ reported shared reading practice.

## Background

Caregiver involvement in children’s early reading development plays a pivotal role in their later reading and scholastic success and usually depends on the parents’ own social, cultural and linguistic capital (Coetzee et al., 2021; Justice et al., 2020). International studies have highlighted the importance of parental involvement in the development of emergent literacy (e.g. Coetzee et al., 2021; Vally et al., 2015). There are many activities that can be utilised to promote emergent literacy and readiness for formal reading instruction, one of which being shared book reading between caregivers and children. Based on such evidence, some South African initiatives have been implemented to promote shared book reading by providing families with books (e.g. Book Dash [www.bookdash.org] and the Mikhulu Trust [www.mikhulutrust.org]). The effective implementation of such initiatives is often encumbered by misconceptions about emergent literacy-related activities. South Africa’s history of inequality and social injustice has resulted in many caregivers having limited access to education and thus presenting with low or absent levels of literacy (Graven, 2016). Research has also shown that many caregivers do not perceive themselves as central partners in their children’s emergent reading development and do not know how to share books with them (Coetzee et al., 2021). This article describes an intervention undertaken with caregivers to promote their shared reading ability. The following sections provide context about children’s literacy development in South Africa and the role of speech-language therapists (SLTs) in shared reading.

## Emergent literacy development and literacy as a social practice in South Africa

The experience when a more knowledgeable other, such as a parent or a teacher uses a combination of targeted strategies, specifically aimed at improving a child’s early language and emergent literacy development, is termed shared book reading (Vally, 2012). This interactive form of sharing books with children has been considered an essential cognitive stimulation activity, which varies widely among cultures and families (Vally, 2012). It is less common in more disadvantaged communities (Fletcher & Reese, 2005; Logan et al., 2019). A study by the South Africa Book Development Council (SABDC, 2011) revealed an absence of reading culture in most low-middle income (LMI) communities in South Africa. Oral literacy is culturally more familiar to many South African caregivers than print literacy and shared book reading. Oral literacy refers to meaning, messages and teachings traditionally conveyed by songs, rituals and oral storytelling rather than by print (Gardner-Neblett & Iruka, 2015). Children’s learning experiences are enriched and enhanced by these forms of knowledge, which are generated within their cultures (Gardner-Neblett & Iruka, 2015). Although shared book reading may not be the sole predictor for emergent literacy development, it has been found to have a distinct facilitative effect on children’s acquisition of language and emergent literacy skills (Cabrera et al., 2020).

## Benefits of shared book reading and the role of the speech-language therapists

The benefits of shared book reading for children’s early language and literacy development have been widely and extensively researched (Coetzee et al., 2021; Shahaeian et al., 2018; Vally et al., 2015). Book sharing in the first six years of a child’s life has been linked with promising (Shahaeian et al., 2018). Substantial enhancements in the language skills of 2-year-old children were observed in a study by Whitehurst et al. (1988) after mothers from middle-class backgrounds had been taught and had improved upon their shared reading techniques. Another study that was conducted by Huebner and Meltzoff (2005) established that a positive effect on children’s language could be attributed to the increase in their parents’ dialogic reading behaviour. Parents who have received instruction and training on how to participate and effectively engage in shared reading strategies have been observed to make greater use of labelling, questioning, commenting and discussing pictures in a story, which ensures a greater chance for exposure to new vocabulary and concepts (Mol et al., 2008). Shared reading skills, experiences and confidence of adults facilitating and mediating literacy development vary greatly. Many adults tend to refrain from sharing books with their children as they lack the knowledge to effectively do so (Tayob & Moonsamy, 2018). The importance of teaching caregivers skills in how to effectively engage in shared reading with young children, especially in settings where there may have been limited opportunity to acquire these skills, highlights the need for emergent literacy interventions that target specifically caregivers.

Despite it being well known that shared reading is an effective vehicle for the promotion of early language and literacy development, a paucity of research has examined the potential benefits of providing caregiver training to achieve this (Dowdall et al., 2019). Concerns surrounding the cost of training and other associated logistical challenges have resulted in few studies describing the effectiveness of these interventions in resource-constrained environments such as South Africa. A study that was conducted by Knauer et al. (2019) in a similar resource-constrained environment in Kenya sets out to investigate the use of dialogic reading training to educate parents about book sharing. The implemented training programme improved the quality of parent–child reading and showed vocabulary gains in children of caregivers with no and/or low literacy. Seven and Goldstein (2020) used a multiple-baseline design to determine whether structured training in dialogic reading and conversational skills impacted upon child language development in four father-child dyads in a resource-poor setting in Turkey. The authors reported interactions and decontextualised talk as study outcomes. A randomised book sharing intervention trial was conducted by Vally et al. (2015) in a rural settlement in South Africa. Children’s early language and literacy development were found to benefit substantially from book sharing intervention, and Vally et al. (2015) surmised that the positive effects that were observed in their study were attributable to improved parents’ mediation skills, which had been developed through training. A study by Coetzee et al. (2021) investigated the impact of a short caregiver training video on the shared reading behaviours of caregivers and children and found a significant improvement in caregiver and children’s shared book reading behaviours after exposure to the training video. The use of training videos as effective and cost-effective intervention is supported by this and other studies (e.g., Lloyd & Robertson, 2012; Hsin & Cigas, 2013). The implementation of training videos as a population-based intervention to promote children’s emergent literacy is in majority world settings is, proves to be promising. The positive outcomes of developing an intervention delivered through the medium of videos demonstrated in these studies served as a scaffold for the current study.

Speech-language therapists are key contributors in promoting children’s emergent literacy because literacy is typically viewed as being built on a foundation of oral language skills. Speech-language therapists facilitate and teach interactive reading behaviours to families, caregivers and teachers. One of their roles is to assist caregivers in creating many high-quality opportunities to participate in emergent literacy activities at home, in daycare and in preschool (Chang & Monaghan, 2019). Speech-language therapists may serve as leaders in designing and implementing literacy programmes such as shared book reading interventions to teach parents and caregivers these interactive reading behaviours (Kaderavek, 2015). Thus, this study set out to understand caregivers’ views about shared reading and how SLTs can use cost-effective early interventions to change these perspectives to promote language and literacy development in South African pre-school children.

The authors endeavoured to answer three research questions:

1. What are caregivers’ pre-intervention perceptions of shared reading?
2. What are the existing barriers to shared reading?
3. Does a training video aid in modifying these attitudes?

## Methodology

### Aim

The study aimed to describe caregivers’ perceptions of shared reading, identify existing barriers to shared reading and determine the impact of a caregiver training video in altering these attitudes.

### Research design

The data for this article formed part of a larger data set for a study that used a non-randomised, comparison group, crossover repeated measures approach as part of a mixed-methods design (Coetzee et al., 2021). In this article, we present the qualitative results obtained from semi-structured interviews with caregivers. All participants were interviewed individually before taking part in the intervention. They were then interviewed again afterwards.

### Participants

Convenience sampling was used. An invitation to participate in the study was extended to caregivers at parent meetings that were held at three separate daycare centres in Orange Farm, Gauteng. Prospective participants had to be a caregiver of a child between 2 and 6 years of age, attending one of the three daycare centres and must have provided signed informed consent. Caregivers with a known visual, cognitive or hearing impairment, as well as minor caregivers, younger than 18 years of age, who did not have signed informed consent from their primary caregiver to participate in the study, were excluded from the study. During the parent meetings at the respective daycare centres, 59 caregivers volunteered to participate. The study process was completed by 40 of these child-caregiver dyads who met the inclusion criteria by attending both appointments. The study comprised a total cohort of 40 participants (*n* = 40). Children in this study ranged in age between 36 and 71 months of age (*M* = 50; s.d. = 9). The cohorts of children who participated in this study (*n* = 40) consisted of 25 who were female (62.5%). Most caregivers were single mothers who reported they were heading their household either with the help of a grandmother or other female support, such as an aunt or an older child. Table 1 provides an overview of the participants.

**TABLE 1:** Demographic information of participating caregivers.

Variable	*n* = 40
**Relationship to child**	
Mother	26
Father	4
Grandparent	4
Sibling	5
Uncle	1
**Education**	
< Grade 12	9
Grade 12	19
Tertiary	12
**Employment status**	
Employed	12
Unemployed	28
**Primary caregiver**	33
**The primary language spoken at home**	
isiZulu	14
Sesotho	20
Other	6
**Estimated time spent with the child per day**	
< 3 hours per day	10
> 3 hours per day	30

### Site of data collection

This study took place at three daycare centres in a semi-rural township called Orange Farm, located south of Johannesburg in Gauteng, South Africa. Orange Farm is a large informal or peri-urban settlement, which is regarded as one of the most geographically isolated communities in Johannesburg. The Orange Farm community is faced with a multitude of challenges including, but not limited to, poverty, high levels of violence, unemployment and crime, limited access to healthcare facilities and lack of basic services, as well as low levels of literacy. Orange Farm is termed semi-rural or peri-urban because of its limited facilities and high levels of unemployment and poverty. All three daycare centres were based in Orange Farm, and the roads leading to them were a combination of dirt roads and poorly maintained tarred roads. Orange Farm has only one small official library located near the Orange Farm Community Centre, which is situated within a 5 km – 10 km radius from each of the three daycare centres. Many residents make use of horse or donkey-drawn carts as modes of transport. The three primary languages most commonly spoken in the Orange Farm area include English, isiZulu and Sesotho (Statistics South Africa, 2018).

## Materials

### Caregiver training video

The 4 min and 30 s caregiver training video (https://www.youtube.com/watch?v=3SlrFzQRKoA) was developed by one of the researchers in English, and a forward or background translation process was then used to translate the video content into isiZulu and Sesotho (Regmi et al., 2010). This video inspired by the parent–teacher training DVD called ‘Read Together, Talk Together’ and was specifically developed and culturally adapted for this study (RTTT; Pearson Early Learning, 2003). The caregiver training video aimed to teach caregivers how to enhance attention to text, promote interactive reading and support comprehension and use literacy strategies effectively. A combination of edited video clips of one of the researchers modelling a shared reading behaviour with a child, narration or explanation and visual caricatures and written bullet points were all combined in the video to maximise the caregivers’ learning experience. The participants were not given access to the caregiver training video on their phones during the study and only watched the training video once during the data collection process. Participants could watch the video more than once if they wished and requested to do so. Other than the training video, no further training was provided by the researchers.

### Books

The books that were chosen for this study were developed by Book Dash. The books that were selected did not necessarily require the caregivers to be competent readers, as all the books primarily comprised colourful pictures and illustrations. Both caregivers and children were thereby free to engage in a meaningful exchange in their own language, irrespective of the caregivers’ level of literacy. Book Dash storybook developers assisted the researchers with the appropriate selection of books and ensured that these were leveled to meet the requirements for the study population (i.e. diversity of languages spoken in South Africa and the geographical context of the study, age of the participants, etc.). All the storybooks were provided in English, Sesotho and isiZulu.

### Semi-structured interview schedules

The researchers developed two interview schedules to include pre- and post-intervention information: the initial semi-structured interview and the semi-structured exit interview schedule. The initial semi-structured interview aimed to obtain demographic information from the participants including age and gender of the primary adult or minor caregiver, age and gender of the child, the caregiver’s highest level of education and occupation, the duration of time that the child has spent in a formal schooling environment, the book-sharing practices at home (if any), reasons for sharing or not sharing books and whether or not books were shared with the parent or caregivers as children. The semi-structured exit interview schedule was intended to obtain insight into the caregiver’s experiences during the project and whether or not they felt that the caregiver training video had changed the way they thought about sharing books with their child, the way that they shared books with their child or children at home and what they had found most beneficial about the intervention.

## Research procedure

The information presented here forms part of a larger study and only the qualitative findings from the interviews will be presented (Coetzee et al., 2021). The larger study used an experimental intervention in the form of a caregiver training video. Data collection took place over a 4-week period with a 2-week duration between the two appointments of each group. The existing perceptions of and barriers to shared reading among caregivers in South Africa were probed in an initial semi-structured interview administered by the researcher in English during the first appointment. Two weeks later, an exit interview was conducted post the caregivers viewing the caregiver training video to determine whether the training video had altered these perceptions. The effects of the video on the caregiver perceptions of shared reading were recorded and analysed. The results were thematically analysed and theoretically linked to the intervention (caregiver training video) being investigated in the study.

### Data analysis

The data were analysed using a combination of content and thematic analysis (Creswell, 2009). Themes were systematically formulated from the data. Themes were tallied as often as the 40 participants mentioned their respective codes, regardless of how often one participant may have mentioned a theme. Thematic analysis took place in three phases, namely coding, of which the purpose was to organise the data; categorisation, in order to distinguish between the various ideas in the data collected and finally, the identification of themes (Braun & Clarke, 2006). To ensure trustworthiness, the interviews were video recorded to allow for playback and review. The researcher and two trained research assistants alternated analysing and crosschecking each other’s analyses.

### Trustworthiness

To ensure trustworthiness, Shenton’s (2004) strategies were implemented. The semi-structured interview questions were piloted in accordance with Shenton’s (2004) strategies, to determine content and face validity. Participants who participated in this study did so voluntary, and hence, only responses from caregivers whose verbal and written consent had been obtained were included in the analysis. Collaborative coding was applied to mitigate any research bias and all authors provided descriptions of both the parameters and the limitations of the study.

### Ethical considerations

Ethical clearance (protocol number: H19/05/04) was obtained from the University of the Witwatersrand Human Research Ethics Committee (non-medical) and the Gauteng Department of Education (GDE) (8/4/4/1/2). All caregivers gave written permission and consent to participate in the study and for their responses to be used in the write up of this study.

## Findings and discussion

Eight main themes emerged from the qualitative analysis. Each of these is discussed in turn below, together with any subthemes that emerged. The themes and subthemes, with verbatim examples from the participants, are presented in Table 2. Themes and subthemes are arranged and discussed according to the aim of the study as well as their occurrence in the initial and exit interview.

**TABLE 2:** Themes and subthemes, with verbatim examples from the participants.

Themes and subthemes from initial semi-structured interview	Explanation	Examples and notes
**Existing perceptions of book sharing**		
1. Absence of a culture of book sharing	Reading is not necessarily an activity that caregivers considered their responsibility and many caregivers appeared unaware of their potential roles in supporting emergent literacy development. Many caregivers did not have a reference of this ever being done with them, and therefore did not see the necessity for sharing books with their own children.	‘Nobody shared books with me when I was young.’ (Participant 10, 30 years, mother) ‘I thought reading a book for my son was boring.’ (Participant 5, 43 years, mother) ‘I remember my mother reading Animal Farm when I was a[*n*] older child. Not when I was little like this one.’ (Participant 13, 25 years, mother) ‘My mum, worked for a [*woman*] who used to give us little books. I remember sitting and trying to read them on my own, but when my mum was finished with her work, she would come and sit with us to read with us. It was nice!’ (Participant 6, 33 years, mother)
2. Reading for education rather than recreation	Reading was viewed as an educational activity that is associated with formal schooling, rather than a recreational activity that can be shared and enjoyed with children.	‘We read the Bible and he must remember the words in the verse. He must repeat them to me later, to remember.’ (Participant 16, 22 years, mother) ‘I think that this [*book sharing*] is something that comes in older years, like grade 1.’ (Participant 23, 29 years, mother)
**Existing barriers to book sharing**		
1. Lack of access to books and reading material	Books are considered a luxury and are not affordable in the majority of households. Limited access to books adversely affects the development of a reading culture.	‘Books? I never think of buying books for her. They [*are*] expensive and I don’t think she likes books.’ (Participant 11, 24 years, mother) ‘I’ve taken her to the library one or two times, but it is far for us to walk and, eish, and the Taxi is expensive.’ (Participant 12, 35 years, father)
2. Perceived limited book sharing skills	Many caregivers are not only unaware of their potential roles in supporting emergent literacy development, but also often feel ill-equipped or unskilled to perform this role.	‘I have no idea how to do that [*share books*].’ (Participant 20, 17 years, sister) ‘I did not know that I can make it fun for her. I did not think that I can make sharing books with her when she is so little, so fun and enjoyable. I thought she is too small for books.’ (Participant 8, 35 years, mother) ‘Reading with her when she is so small honestly never crossed my mind.’ (Participant 4, 14 years, sister) ‘I have been working with children all my life and never would I have thought of reading a book with my 3-year-old. I did not think it was possible.’ (Participant 23, 29 years, mother)
3. Limited time	The majority of South African households rely on a single income. Because of many households not having access to their own modes of transport, a great deal of time is spent travelling to and from their places of work by public transport. This means that any remaining time is spent on fulfilling fundamental needs such as cooking, cleaning etc.	‘My busy lifestyle makes sharing books difficult. It has not been a priority for me.’ (Participant 3, 33 years, mother) ‘I am a working mum. I work in the day and sometimes in the weekend. When I come home, I do the other things too. I must cook and wash. Eish, it’s a lot.’ (Participant 9, 34 years, mother)
**Perceptions of shared reading post intervention**		
1. Shared reading as a vehicle for change	Many caregivers were genuinely unaware that a simple activity such as sharing a picture book with their child, could be such an effective vehicle for change. Many caregivers were surprised by the impact this had had on their children’s communication.	‘I never thought of that [*sharing books*]. I think there is a lack of information about that and how much of a difference it can make to our children.’ (Participant 20, 17 years, sister) ‘I did not see any reason to share books with [*my child*]. I did not see the benefit until now. Everything has changed.’ (Participant 12, 35 years, mother) ‘I now know that it is important to share books with children when they are young.’ (Participant 8, 35 years, mother) ‘I did not know much about reading. I thought it was just about learning words and boring like in school … I did not know you can make it fun for her. Not that maybe you can make some funny noise to get her interest.’ (Participant 1, 32 years, mother)
2. Improved relationships	Book sharing promoted a perceived improvement in parenting capabilities and improved relationships between caregivers and children.	‘It [*the project*] change a lot how I feel about sharing books with her. We have more time together and we bond a lot.’ (Participant 23, 29 years, mother) ‘Best part is how to communicate with my child and connect with my child.’ (Participant 4, 14 years, sister) ‘Sharing a storybook with your child – it brings so much connection between me and my child, and I must show some reaction so that the child can understand what the story is all about … It helps to have a good communication with our kids.’ (Participant 13, 25 years, mother) ‘She wants me to read with her all the time now.’ (Participant 17, 39 years, mother) ‘Sometimes she forgets to go with other kids and play and then she say to me “Mama! Can we read that book?”’ (Participant 11, 24 years, mother) ‘It has helping me to interact with him and him to interact with me. It is so nice.’ (Participant 27, 50 years, grandmother)
3. Increased frequency of book sharing and the child as an active participant in book sharing	Book sharing allowed for, and encouraged, both the caregiver and the child to actively participate in the reading process. Both child and caregiver were encouraged to become equal contributors.	‘Lately, since our first appointment with you, we have been sharing all the time. But it’s not me sharing with her; it’s her sharing with me. She makes me share books with her now.’ (Participant 13, 40 years, mother) ‘Every day, we share now. She wants to share books and asks to share books with me.’ (Participant 5, 43 years, mother). ‘She insists on holding the book herself and turning the pages. She is convinced that she is reading me the story.’ (Participant 1, 32 years, mother) ‘Now when we read, she wants to also read and imitate and now she also points to pictures and talk about them or make up her own stories for the pictures.’ (Participant 22, 25 years, mother)
4. Increased verbal output among the children	Responsive and reciprocal interaction between caregivers and young children promotes the acquisition of new vocabulary and the development of linguistic skills.	‘It is so amazing to hear her talk so much and how much she actually knows.’ (Participant 1, 32 years, mother) ‘She does not let me hold the book anymore when we share the book. She takes it from me and sits like me when I read to her and then she pretends to read the story to me.’ (Participant 17, 39 years, mother)
5. The benefits of age appropriate resources	Books that are easy to read and contained many colourful pictures, made it easier to tell a story or make up a story, even if caregivers did not read the words.	‘The books were really great. The writing and the English were easy to read, and the pictures were colourful and visible and easy.’ (Participant 4, 33 years, mother) ‘I like the books you give us. They are nice for a younger child. They are child-friendly and relevant to a child’s life. They are at the level of the child.’ (Participant 6, 33 years, mother)
6. The effectiveness of the video intervention format	The caregiver training video allowed many caregivers to feel more comfortable, as this was perceived to be non-threatening, and they did not feel in danger of being judged by a teacher or instructor.	‘It helped me to see the interactions between you and the children in the video. Like a model or … [*uhm*] … like examples, you know?’ (Participant 12, 35 years, father) ‘To see how you interacted with the children made it easier for the children to share a book with you. Seeing you do that taught me how to make her understand what is happening in the book and to make it more fun.’ (Participant 13, 40 years, mother)

### Existing perceptions of book sharing

#### Absence of a culture of book sharing

The majority of participants in this study stated that they did not share books with their 3- to 6-year-old children, even those who indicated that they owned some form of reading material at home. Most believed sharing and reading books was an activity that was the role of the school and schoolteachers and reserved for their older, school-going children rather than their pre-school child. Research supports the observation that reading is not necessarily an activity that caregivers consider their responsibility and parents in LMI groups in South Africa are often unaware of their potential roles in supporting emergent literacy development (Seden, 2008). The few participants who reported sharing books with their children described sharing colouring books, tracing books, comics or school setwork books belonging to an older sibling. The most commonly mentioned reason for the absence of a culture of book sharing – leading to a subtheme that emerged from this study – was the absence of a culture of book sharing and reading experiences in their own homes when they were children. Many participants reported that they had no recollection of a caregiver ever having shared a book or read a book with or to them. ‘I have no memories of [anyone] sharing books with me as a child. This was not done at home’ (Participant 15, 20 years, mother). The absence of a culture of book sharing can, at least in part, be ascribed to the fact that most of the parents of the participants in this study were born and raised between 1948 and 1994 – the Apartheid era in South Africa. The capacity of primary caregivers in South Africa (especially those living in rural communities) to provide reading materials for their children is often limited and adversely affected by poverty, limited knowledge and literacy skills (Makunga et al., 2017). Of the 40 caregivers who participated in this study, only two reported sharing books with their own caregivers during their childhood. Children’s reading preferences are developed in social contexts and reinforced by people in their immediate environment. A study conducted by Pfost et al. (2016) assessed the attitudes of 380-nineth grade students to reading. The mothers of these students were asked to provide comparable information about their own reading behaviours, attitudes and habits. Results obtained from the study revealed that caregiver attitudes and reading behaviours do impact children’s attitudes towards reading and their reading behaviours.

#### Reading for education rather than recreation

Participants also mentioned that they viewed reading and book-related activities as educational rather than recreational. ‘I think that this [*book sharing*] is something that comes in older years, like grade 1.’ (Participant 23, 29 years, mother). Research suggests that this is a notion shared by many South Africans (Haese et al., 2018). When asked whether they shared books with their children at home, many caregivers mentioned that they would read religious texts to the children, which they would then be required to recite.

### Existing barriers to book sharing

#### Lack of access to books and reading material and perceived limited book-sharing skills

The most prominent barrier to sharing books with their children at home was the lack of access to books and reading materials. The SABDC (2007) national survey found that LMI households are less likely to have books. Ninety-seven percent of households in sub-Saharan Africa has two (or fewer) children’s books (United Nations International Children’s Emergency Fund [UNICEF], 2017). Limited access to resources in rural settlements, such as Orange Farm, is a daily reality for many families. Books are considered a luxury and are not affordable in most households. ‘I don’t have children’s books. They are too expensive’ (Participant 5, 43 years, mother). Limited access to books adversely affects the development of a reading culture. Only one participant mentioned having visited a library with her child on one or two occasions. According to Pretorius (2015), an integral part of developing a culture of reading is access to age-appropriate books. Children require access to books and a skillful model such as a parent or an older sibling to facilitate a love for reading (Pretorius, 2015). Many caregivers are not only unaware of their potential roles in supporting emerged literacy development but also often reported feeling ill-equipped or unskilled to perform this task.

#### Limited time

Many caregivers pinpointed a lack of time as being a significant factor that inhibited them from sharing books with their children. ‘I come home late from work. I work for long hours and then I am too tired when I come home’ (Participant 26, 25 years, mother). Another participant mentioned that she was often too tired after a long day at work. An estimated 40% of South African households are headed by a single parent, of which 40% are headed by a female. Female-headed households face an increased risk of poverty as a result of their limited access to social and economic resources and the additional costs of childrearing (Department of Social Development, 2013). The prioritisation of chores may therefore be a reason why many single-parent households rely on their eldest child to share some of the child-rearing responsibilities and why older siblings accompanied some of the children in this study.

### Perceptions of shared reading post intervention

#### Shared reading as a vehicle for change and improved relationships

The reading context and attitudes of caregivers after exposure to the caregiver training video appeared to change. Most caregivers reported altered shared reading behaviours and that their view of sharing books with young children had shifted after watching the video. This change in attitude was multi-faceted – it applied to the caregivers’ attitudes regarding the importance of book sharing and their improved relationship with their children and new skills development:

‘It [*the project*] has changed the mind-set I once had of not having an interest of sharing a book with a young child and it made me eager to share books more with my young ones.’ (Participant 12, 35 years, father).

Parental involvement at an early age is a critical component of reading skills development (Sukhram & Hsu, 2012). Caregivers felt encouraged and positively reinforced by the positive responses they felt they elicited from their children during shared reading. Research has shown that book sharing can promote a perceived improvement in parenting capabilities (Seden, 2008). This coincides with the findings of Lessing and Odendaal (2004) that the aspects contributing to the success of such an intervention are the physical presence of the caregivers, the positive reinforcement from the caregivers, improved self-confidence among the children and the caregivers and quality time spent together.

#### Increased frequency of book sharing

Many participants emphasised that the frequency of book-sharing activities had increased within the household and that the children often initiated this:

‘We share books much more now. Even when I forgot, she shows me that we haven’t read today. [*I*] did not think that I could make reading books with her so enjoyable and fun.’ (Participant 15, 20 years, mother)

Many South Africans consider reading as educational rather than recreational, and this perception was changed over the course of the study. Participants admitted to having considered reading and sharing books to be a boring and difficult activity. They had not been aware of ways to make it more interesting and intriguing for the children. This observation supports the notion of transgenerational communication of assumptions regarding literacy in that the caregiver/s’ own attitudes towards schooling and literacy were affected by their own school experiences (Reese et al., 2012).

#### The child as an active participant in book sharing

Book sharing allows for – and encourages – both the caregiver and the child to actively participate in the reading process. Both child and parent are encouraged to become equal contributors. Several participants mentioned that their children had begun insisting on holding the book and leading the interaction after having observed their caregiver share a book with them. Caregivers reported that, as they continued sharing books with their children, their children became more confident and would begin holding the books, turning the pages and narrating the story more independently:

‘Before, I just read the words and I quickly turn the pages to finish the book. Now, I take time and I don’t just read the words. I let her talk and point the pictures and talk about the pictures.’ (Participant 2, 25 years, father)

#### Increased verbal output among children

Participants reported engaging more in collaborative talk. They learned to make use of this form of communication with their children to assist them with comprehension and, importantly, facilitate the enjoyment of the reading process. These findings relate to Bandura’s (1977) social learning theory, which suggests that children observe their role models in their immediate environment and study their behaviours with the potential for later imitation. The children’s desire and resulting eagerness to engage in independent reading also indicate a sense of personal mastery that will ultimately aid in fostering an intrinsic motivation for reading (Fawson & Moore, 1999). Responsive and reciprocal interaction between caregivers and young children promotes the acquisition of new vocabulary and the development of linguistic skills (Knauer Jakiela, Ozier, Aboud & Fernald, 2018). Many participants also mentioned that their children had become more verbal during the project:

‘She does not stop talking now. She asks me questions about everything and wants to know the reasons for everything that is happening in the book. Sometimes I don’t know. We can sit for hours and speak about things that are happening in the book.’ (Participant 11, 24 years, mother)

#### The benefits of age-appropriate resources

All participants reported that the picture books used in this study had an impact on the reading practices in their homes. Participants repeatedly mentioned that the books were easy to read and contained plenty of pictures, which made it easy to tell a story even if they did not read the words:

‘I like that they [*the books*] have many pictures and they are child friendly. Even though you don’t read all the words, you can grasp the story from the pictures or make your own story.’ (Participant 3, 33 years, mother)

The participants made use of the books in a multilingual fashion. Although the researcher had provided each of the books in English, isiZulu and Sesotho, the written language or story text did not affect the language of narration during shared reading. All the participants were observed code-switching at least once during their interactive shared reading session with their child. Along with isiZulu, Sesotho, isiXhosa and Sepedi, English was also used consistently during the interactions. Many of the caregivers elected to share the English version of a book with their child but would narrate the entire book in their home language. The books allowed for the shared reading interactions between the caregivers and the children to take place in the language of the participant’s choice. This concept of scaffolding coincides with a growing body of work within South Africa on ‘translanguaging’ (Canagarajah, 2011; Makalela, 2015). Translanguaging assumes that language practice involves a complex inter-play of communicative strategies including, for example, code-switching between languages during an interactive reading activity. This form of scaffolding needs to be encouraged during these shared book-reading interactions to aid children’s expressive and receptive language development in English and the child’s home language. Bilingual transitional models maintain that a child must first develop cognition in the medium of their first language before they are able to gain the necessary skills for the acquisition of a second language (Canagarajah, 2011; Makalela, 2015). These debates have implications for language policy in South Africa, and evidence from this study suggests that home language has an important role to play in the development of early reading skills. Vygotsky’s (1978) notion of ZPD is also relevant to second language acquisition. If a child has not attained a comfortable level of oral language proficiency and does not comprehend the fundamental principles of emergent literacy in their first language, then academic mastery of a second language will be beyond the ZPD. The use of multiple languages and ‘translanguaging’ during shared reading activities builds and strengthens the development of cognitive skills in children (Peng & Kievit, 2020).

#### The effectiveness of the video intervention format

Several participants mentioned that they appreciated and enjoyed seeing the shared reading technique being modeled in the caregiver training video:

‘It [*the video*] guided me on how to interact. I have learned how now to wait and let her interact. So actually, if I did not see the video, I was going to be dominant from beginning to end.’ (Participant 14, 33 years, mother)

Most caregivers emphasised that they had enjoyed the caregiver training video as a vehicle of learning and teaching. Many reported that they felt comfortable learning via a video format, as this was perceived to be non-threatening, and they did not feel in danger of being judged by a teacher or instructor.

## Conclusion

This article explored caregivers’ perceptions of shared reading before and after an intervention to improve shared reading practices among caregivers. Targeting early language and literacy development, and thereby contributing to reducing educational inequality, is a potential advantage of a parent-centred shared reading intervention. The overall changes elicited in attitudes towards shared reading and the increase in shared reading evident among caregiver suggest that this caregiver training video provides a promising and effective early intervention worth implementing in LMI households in countries like South Africa. Positive effects of a caregiver training video on the shared book reading practices and relationship between caregivers and their children are supported by this study. However, the cultural context of South Africa is complex, and factors impacting emergent literacy development are multi-faceted. There is still a great deal of work to be done before an intervention model is developed that accommodates the diversity of cultures that exist in South Africa.

This study has some limitations in that the cohort recruited was relatively small, and results can therefore not easily be generalised as contextual factors will differ. Furthermore, the children who participated in this study were of a large age range, which complicates the interpretation of findings about specific shared reading behaviours for specific age groups as emergent literacy skills develop across a continuum. Similarly, the same applies to the large age range of caregivers as this introduced additional variables that necessitate cautious interpretation of results. However, this situation reflects typical communities in the majority world countries. The role of the reading partner, such as siblings versus parents, needs to be further researched as each group will bring a diversity of life experiences, strengths and weaknesses to their interactions and reading behaviours. The results obtained in this study report on the short-term perceptions of caregivers. Further studies would need to investigate the sustainability of reported new behaviours and what minimum intervention dosage yields the most long-lasting results.

The absence of book-sharing practices in South Africa can be attributed to a myriad of barriers related to past and current challenges of LMI families in South Africa today. However, results obtained also showed the promising change and impact that a caregiver training video can have on the attitudes of caregivers towards book sharing and the power to modify behaviour to improve the quality of caregiver-child shared reading interactions. Making intervention and academic materials available in the 11 official languages spoken in South Africa is therefore of critical importance and highly recommended. Early interventionists play a pivotal role in the development, implementation and promotion of such training programmes. Speech-language therapists have a key role in the promotion of early literacy skills particularly in children who may be at risk for developing literacy-related learning difficulties. Speech-language therapists may help to prevent, identify and provide suitable interventions for these children and collaborate with families, caregivers and teachers to ensure that children are provided with opportunities to participate in emergent literacy activities within their immediate environments. Speech-language therapists need to endeavour to incorporate existing cultural practices and foster a responsiveness to their context. Speech-language therapists who are based in LMI countries such as South Africa should be encouraged to contribute to the development of further emergent literacy interventions that are easily disseminated and cost-effective and require minimal trained personnel and expertise. Speech-language therapist’s also need to recognise the importance of caregiver training to foster the development of emergent literacy skills.
